# Small intestinal bacterial overgrowth as a cause of protracted wound healing and vitamin D deficiency in a spinal cord injured patient with a sacral pressure sore: a case report

**DOI:** 10.1186/s12876-020-01423-8

**Published:** 2020-08-24

**Authors:** Yoshitaka Kubota, Hidekazu Nagano, Kentaro Ishii, Takashi Kono, Satomi Kono, Shinsuke Akita, Nobuyuki Mitsukawa, Tomoaki Tanaka

**Affiliations:** 1 Department of Plastic Surgery, Chiba University, 1-8-1, Inohana, Chuo-ku, Chiba-city, Chiba #260-8670 Japan; 2 Department of Molecular Diagnosis, Chiba University, 1-8-1, Inohana, Chuo-ku, Chiba-city, Chiba #260-8670 Japan; 3 Department of Plastic Surgery, Chiba Emergency Medical Center, 3-32-1, Isobe, Mihama-ku, Chiba #261-0012 Japan

**Keywords:** Pressure wound, Small intestinal bacterial overgrowth, Spinal cord injury, Malnutrition, Wound healing, Case report

## Abstract

**Background:**

Pressure sores are sometimes refractory to treatment, often due to malnutrition. Small intestinal bacterial overgrowth (SIBO) obstructs absorption in the digestive tract and causes malnutrition. However, little is known about the association between pressure sore wound healing and SIBO. Here, we report a case of a patient with a refractory sacral pressure sore and SIBO.

**Case presentation:**

A 66-year-old woman who was spinal cord injured 14 years before visiting our hospital presented with the chief complaint of a sacral pressure sore, 10.0 × 6.5 cm in size, which was refractory to treatment. Physical examination showed abdominal distension and emaciation, with a body mass index of 15. Further examination revealed elevated serum alkaline phosphatase (1260 U/L), bilateral tibial fracture, multiple rib fracture, and osteoporosis. We diagnosed the patient with osteomalacia with vitamin D deficiency. Despite oral supplementation, serum levels of calcium, phosphorous, and vitamin D remained low. Also, despite concentrative wound therapy for the sacral pressure sore by plastic surgeons, no wound healing was achieved. Due to a suspicion of disturbances in nutrient absorption, we performed bacterial examination of collected gastric and duodenal fluid, which showed high numbers of bacteria in gastric content (10^4^ *E. coli*, 10^5^ *Streptococcus* species, and 10^5^ *Neisseria* species) and duodenal content (10^6^ *E. coli*, 10^4^ *Candida glabrata*). Therefore, we diagnosed the patient with SIBO and started selective decontamination of the digestive tract using polymyxin B sulfate and amphotericin B. After starting treatment for SIBO, the sacral pressure sore began to heal and was nearly healed after 285 days. The patient’s serum levels of calcium, phosphorous, vitamin D, and other fat-soluble vitamins also gradually increased after starting treatment for SIBO.

**Conclusion:**

We report a case of a patient with a refractory sacral pressure sore that healed after starting treatment for SIBO. We conclude that SIBO may be an overlooked cause of malnutrition and poor wound healing in patients with chronic pressure sores.

## Background

Pressure sores in patients with spinal cord injury (SCI) are sometimes refractory to treatment. Chronic gastrointestinal symptoms are also frequently seen in patients with SCI [1, 2], and malnutrition caused by decreased gastrointestinal motility in SCI patients is a major cause and exacerbating factor of pressure sores. Evaluation of nutritional status in patients with pressure sores is essential [3], as nutritional intervention can be a valuable treatment option for pressure sores. However, small intestinal bacterial overgrowth (SIBO) is rarely considered in the evaluation of malnutrition in SCI patients with pressure sores. SIBO is defined as the presence of more than 1 × 10^5^ colony forming units (CFU)/mL of bacteria or any amount of *E. coli* in the proximal small bowel content [4]. Here, we report the case of an SCI patient with a refractory sacral pressure sore that healed after starting treatment for SIBO. To the best of our knowledge, this is the first report of an association between a pressure sore and SIBO.

## Case presentation

A 66-year-old woman visited our hospital for the purpose of treating her sacral pressure sore (day 0), which she developed 8 months prior due to bed rest during treatment of a left humeral fracture in another hospital. She had paraplegia as well as bladder and rectal disturbance due to SCI at the fourth lumbar level (L4) caused by a suicidal jump in response to paranoid delusions at 52 years of age. Spinal fusion surgery and cystostomy were performed early after SCI. Otherwise, she had a history of hysterectomy due to uterine cancer at 31 years of age, lymphaticovenular anastomosis as a treatment for post-hysterectomy lymphedema in the bilateral lower extremities at 50 years of age, and cholecystectomy at 60 years of age.

**Fig. 1:**
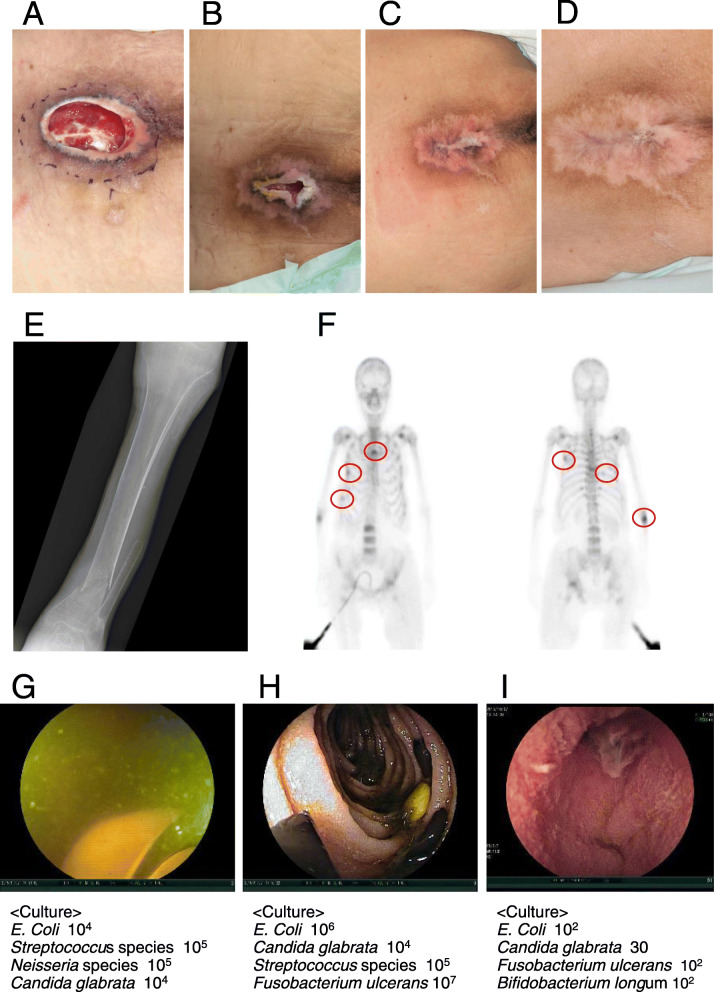
Patient images. **a**, **b**, **c**, and **d** Sacral pressure sore. **a** Day 0: sore 10.0 × 6.5 cm in size with an entrance of 6.0 × 3.5 cm; DESIGN-R score was D3 e3 s8 i0 g3 N3 P24 with a total score of 44. **b** Day 405 (i.e., 285 days after starting SDD for treating SIBO): reduced size of sore; DESIGN-R score was D3 e3 s3 i0 g1 n0 p0 with a total score of 10. **c** Day 742 (i.e., 617 days after staring SDD): healed sore; DESIGN-R score was d0 e0 s0 i0 g0 n0 p0 with a total score of 0. **d** Day 1057 (i.e., 932 days after staring SDD): no recurrence of the sore. **e** and **f** Osteoporosis and multiple fractures. **e** X-ray showing left tibial fracture. **f** Tc-99 m bone scan showing accumulation in multiple ribs, vertebrae, and right ulna. **g**, **h**, and **i** Endoscopic examination and results of bacterial culture of the upper digestive tract. All stomach, duodenum, and proximal jejunum samples were positive for *E. coli*. **g** Stomach. Food residue can be seen. Acid level was decreased to pH 7.0. **h** Duodenum. Food residue is evident. **i** Proximal jejunum. Flat villi and a jejunal ulcer are observed

When she visited our hospital, she was taking the following oral medicines: propiverine hydrochloride, vitamin B_12_, etizolam, flunitrazepam, sodium bicarbonate anhydrous monobasic sodium phosphate mixture, *Clostridium butyricum* tablets, sodium risedronate hydrate, rebamipide, sodium ferrous citrate, fursultiamine hydrochloride, alfacalcidol, and potassium L-asparate. She did not take proton pump inhibitors (PPI). Her vital signs were as follows: body temperature of 36.9 °C, low blood pressure of 83/50 mmHg, pulse rate of 82 bpm, and respiratory rate of 12 per min. Physical examination showed abdominal distension, emaciation with a body mass index of 15, and a sacral pressure sore 10.0 × 6.5 cm in size including a pocket entrance of 6.0 × 3.5 cm (Fig. 1a). Most of the surface of the pressure sore was covered by granulation. Our evaluation of the pressure sore with DESIGN-R [5] was D3 e3 s8 i0 g3 N3 P24, with a total score of 44 (Table 1).

**Table 1:** DESIGN-R assessment tool for pressure sore. (Reprinted with permission from John Wiley and Sons. In: Matsui et al., Development of the DESIGN-R with an observational study: an absolute evaluation tool for monitoring pressure ulcer wound healing. *Wound Repair Regen* 2011, 19(3):309–315)

**Depth**					
**d**	**0**	No particular skin lesion and no redness	**D**	**3**	Lesion extends into the subcutaneous tissue
	**1**	Persistent redness		**4**	Lesion extends to the muscle, tendon, and bone
	**2**	Lesion extends into dermis		**5**	Lesion extends into the articular or body cavity
				**U**	It is impossible to measure the depth
**Exudate**					
**e**	**0**	None	**E**	**6**	Heavy: requires dressing change more than twice a day
	**1**	Slight: does not require daily dressing change			
	**3**	Moderate: requires daily dressing change			
**Size**					
**s**	**0**	None	**S**	**15**	100 cm^2^ or larger
	**3**	Smaller than 4 cm^2^			
	**6**	4 cm^2^ or larger, but smaller than 16 cm^2^			
	**8**	16 cm^2^ or larger, but smaller than 36 cm^2^			
	**9**	36 cm^2^ or larger, but smaller than 64 cm^2^			
	**12**	64 cm^2^ or larger, but smaller than 100 cm^2^			
**Inflammation/Infection**					
**i**	**0**	None	**I**	**3**	Clear signs of local infection (e.g., inflammation, pus, and foul smell)
	**1**	Signs of inflammation (fever, redness, swelling, and pain around the wound)		**9**	Systemic impact, such as fever
**Granulation tissue**					
**g**	**0**	Granulation cannot be assessed because the wound is healed or too shallow	**G**	**4**	Healthy granulation tissue occupies 10% or more, but less than 50%
	**1**	Healthy granulation tissue occupies 90% or more		**5**	Healthy granulation tissue occupies less than 10%
	**3**	Healthy granulation tissue occupies 50% or more, but less than 90%		**6**	No healthy granulation tissue exists
**Necrotic tissue**					
**n**	**0**	None	**N**	**3**	Soft necrotic tissue exists
				**6**	Hard and thick necrotic tissue is attached to the wound
**Pocket**					
**p**	**0**	None	**P**	**6**	Smaller than 4 cm^2^
				**9**	4 cm^2^ or larger, but smaller than 16 cm^2^
				**12**	16 cm^2^ or larger, but smaller than 36 cm^2^
				**24**	36 cm^2^ or larger

Bacterial culture examination of the pressure sore showed *Corynebacterium striatum* and methicillin-resistant *Staphylococcus aureus*. Laboratory data showed an elevated serum alkaline phosphatase level of 1260 U/L and low serum levels of hemoglobin 11.9 g/dL, albumin 2.8 g/dL, calcium 7.8 mg/dL, and zinc 51 μg/dL. On day 22, we observed a sudden decrease of hemoglobin to 6.4 g/dL with a positive fecal occult blood test, bilateral pleural effusion on chest x-ray, and serum albumin level of 2.3 g/dL. Upper gastrointestinal endoscopy showed a gastric ulcer at H2 stage.

As a result of searching for the cause of alkaline phosphatase elevation, bilateral tibial fracture, multiple rib fracture, and osteoporosis were found (Fig. 1e and f). Femoral bone density was 17% of the young adult mean. A low serum inorganic phosphorous level was found (Table 2), along with a low serum level of 25-hydroxy vitamin D_3_ (25(OH)VitD_3_) below the detection limit and elevated level of parathyroid hormone (Table 3). Levels of other fat-soluble vitamins were also low: vitamin A < 5 μIU/dL, vitamin K_1_ < 0.05 ng/dL, and vitamin E 0.21 mg/dL. Examination using ultrasound and computed tomography showed normal thyroid and parathyroid glands. Basing on these finding, we diagnosed osteomalacia with vitamin D deficiency.

**Table 2:** Laboratory data before starting supplementation with vitamin D

WBC	3500	/μL
RBC	332	10^4/μL
Hb	9.5	g/dL
Plt	19.2	10^4/μL
Total Protein	5.3	g/dL
Albumin	2.9	g/dL
AST	40	U/L
ALT	10	U/L
γGTP	7	U/L
LDH	527	U/L
ALP	1225	U/L
Ch-E	119	U/L
CK	24	U/L
Amy	24	U/L
BUN	13	mg/dL
Creatinine	0.31	mg/dL
eGFR	155.1	mL/min/1.73 m2
Na	138	mEq/L
K	5.5	mEq/L
Ca	7.8	mg/dL
iP	1.5	mg/dL
Mg	2.4	mg/dL
Fe	72	μg/dL
Zn	50	μg/dL
UIBC	188	μg/dL
Ferritin	43.7	μg/dL
Erythropoietin	82.9	μIU/mL
Total Cholesterol	95	mg/dL
Triglyceride	87	mg/dL
HDL-Cholesterol	34	mg/dL
LDL-Cholesterol	40	mg/dL
PT-INR	1.01	
APTT	33.2	second
ACTH	20.9	pg/mL
Cortisol	9.9	μg/dL
TSH	5.179	μIU/mL
FT3	1.5	pg/mL
FT4	0.85	ng/dL

**Table 3:** Vitamins and bone metabolism markers before starting supplementation of vitamin D

			(normal range)
Vitamin A	< 5	IU/dL	97–316
Vitamin K_1_	< 0.05	ng/mL	0.15–1.25
Vitamin K_2_	< 0.05	ng/mL	< 0.1
Vitamin E	0.21	mg/dL	0.75–1.41
1,25(OH)2 Vitamin D	20.3	pg/mL	20.0–60.0
25(OH) Vitamin D_3_	< 5	pg/mL	7–41
Retinol binding protein	0.7	mg/dL	1.9–4.6
Vitamin B1	134	ng/mL	24–66
Vitamin B12	2320	pg/mL	180–914
Nicotinic acid	4.4	μg/mL	4.7–7.9
Folic acid	18.7	ng/mL	> 4.0
TRACP-5b	1490	μU/mL	120–420
NTx	1285	nmolBCE/L	14.3–89.0
Bone type ALP	123	μg/L	3.8–22.6
Intact P1NP	181	μg/L	14.9–68.8
Osteocalcin	2.9	ng/mL	14.2–54.8
intact PTH	650	pg/mL	10–65
PTHrP	< 1.1	pmol/L	< 1.0
FGF23	Below the detection limit	pg/mL	19.9–52.9
%TRP	87.8	%	81–90
TmP/GFR	1.414	mg/dL	2.0–3.4

On day 61, oral supplementation of calcium, phosphorous, and vitamin D was started. Despite supplementation, serum levels of calcium, phosphorous, and 25(OH)VitD_3_ on day 89 showed poor improvement: calcium 7.5 mg/dL, phosphorous 2.4 mg/dL, and 25(OH)VitD_3_ below the detection limit.

**Fig. 2:**
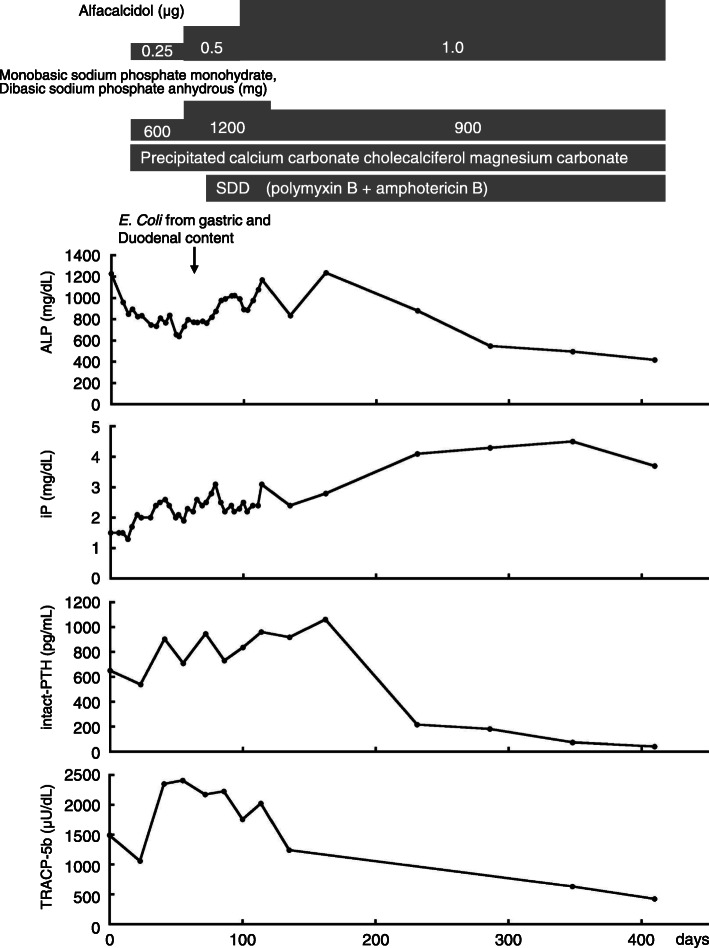
Bone metabolism markers. After starting SDD, levels of bone metabolism markers gradually improved

On day 117, we performed bacterial examination of collected gastric and duodenal fluid with suspicion of a disturbance in absorption, which showed elevated numbers of bacteria in gastric content (10^4^ *E. coli*, 10^5^ *Streptococcus* species, and 10^5^ *Neisseria* species) and duodenal content (10^6^ *E. coli*, 10^4^ *Candida glabrata*) (Fig. 1g, h, and i). Therefore, we diagnosed SIBO. On day 125, we started selective decontamination of the digestive tract (SDD) using oral administration of polymyxin B sulfate, 2.5 million units, daily and oral administration of amphotericin B, 300 mg, daily. Before starting SDD, the pressure sore was refractory to multiple methods of wound treatment, including depressurization, irrigation, debridement, ointment, basic fibroblast growth factor, and negative pressure wound therapy. After starting SDD, the pressure sore began to heal. On day 410 (i.e., 285 days after starting SDD), the pressure sore DESIGN-R score was D3 e3 s3 i0 g1 n0 p0 with a total score of 10 (Fig. 1b). Regarding the nutritional status, the serum albumin level increased from 1.8 g/dL, just before starting SDD, to 3.2 g/dL at 285 days after starting SDD. Also, the hemoglobin level increased to 12.1 g/dL and the serum zinc level increased to 67 μg/dL. Serum levels of calcium, phosphorous, vitamin D, and other fat-soluble vitamins also gradually increased (Fig. 2 and Table 4). A repeat diagnostic bacterial examination of the upper digestive tract contents, for SIBO, was not performed because of obvious improvements in most of the laboratory data. There were no adverse effects of SDD, such as antibiotic-associated diarrhea. In contrast, the patient presented with constipation that was noted before starting SDD. The sacral pressure sore was completely healed on day 742 (i.e., 617 days after starting SDD), with a DESIGN-R score of d0 e0 s0 i0 g0 n0 p0 and total score of 0 (Fig. 1c). In addition, the patient showed improved nutritional status, and had a serum albumin level of 3.4 g/dL. We successfully reduced the dose of polymyxin B from 2.5 to 1.5 million units daily, similarly the dose of amphotericin B was reduced from 300 to 200 mg daily on day 904 (i.e., 779 days after starting SDD) without any signs of SIBO recurrence. There was no recurrence of the sacral pressure sore with a serum albumin level of 3.5 g/dL on day 1057 (i.e., 932 days after staring SDD) (Fig. 1d). On day 1493, we successfully ended the use of amphotericin B; however, the use of polymyxin B at 1.5 million units per day continued. On day 1815 (i.e., 1690 days after starting SDD), while still using polymyxin B at 1.5 million per day, the serum albumin level was 3.9 g/dL, the hemoglobin level was 13.9 g/dL, and the serum zinc level was 114 μg/dL. There were no signs of SIBO recurrence or the sacral pressure sore.

**Table 4:** Vitamins and trace elements before and after supplementation and selective digestive decontamination (SDD)

	Before supplementation	At the time SDD started	56 days after starting SDD	346 days after starting SDD
Vitamin K_1_ (ng/mL)	<0.05	0.96	0.86	N/A
Vitamin K_2_ (ng/mL)	<0.05	0.38	0.47	N/A
Vitamin E (mg/mL)	0.21	0.33	0.34	N/A
1,25(OH)_2_ Vitamin D (pg/mL)	20.3	29	40.5	76.8
25(OH) Vitamin D_3_ (pg/mL)	<5	<5	10	17
Nicotinic acid (μg/mL)	4.4	4.5	5.3	7.3
Mg (mg/dL)	2.4	2	2.2	2.4
Fe (μg/dL)	72	43	33	58

## Discussion and conclusions

We report the case of a patient whose sacral pressure sore and osteoporosis were improved by treatment for SIBO. Although nutrition status is known to be important for the healing of pressure sores, SIBO is rarely checked as a cause of malnutrition in patients with pressure sores. However, SIBO is a potential cause of malnutrition in patients with SCI due to decreased intestinal motility resulting from autonomic disturbances and reduced physical activity [6]. SCI is also a risk factor for pressure sores [7]. However, to the best of our knowledge, there are no previous reports of an association between pressure sores and SIBO. Thus, our case draws attention to the fact that SIBO can be an overlooked cause of poor wound healing during the treatment of pressure sores.

SIBO was first reported by Vantrappen et al. as an increased concentration of ^14^CO_2_ in a bile acid breath test for patients with an absent interdigestive motor complex [8]. Today, consensus diagnostic criteria for SIBO are the presence of more than 1 × 10^5^ CFU/mL of bacteria or any amount of *E. coli* in the proximal small bowel content [4]. Relatively little is known about commensals inhabiting the small intestine, mainly due to the limited accessibility of this environment for microbiological analysis [9]. In the healthy state, the numbers of intestinal bacteria range from 10^2^ to 10^5^ CFU/mL and mainly include gram-negative and gram-positive aerobes, such as *Streptococcus*, *Lactobacillus*, and *Bacteroides* species [10]. Regarding the amount of bacteria in proximal jejunal aspiration, Khoshini et al. report that normal subjects rarely exceed 1 × 10^3^ CFU/mL and therefore proposed more than 1 × 10^3^ CFU/mL coliform bacteria as the threshold for SIBO [11]. In our case, 10^6^ CFU/mL *E. coli* existed in duodenal content, and 10^4^ CFU/mL *E. coli* existed in gastric content, which met the traditional diagnostic criteria of SIBO.

Other diagnostic methods for SIBO are breath tests using hydrogen or hydrogen methane with lactulose or glucose [10]. Breath tests have clinical utility for diagnosing SIBO because they are less invasive than obtaining proximal small bowel content. However, there are no standardized criteria for diagnosing SIBO using breath tests [10]. We did not perform a breath test in our case study.

Despite no previous reports of an association between unhealed pressure sores and SIBO, nutritional status is known to be important for the healing of pressure sores [3]. In our case, the sacral pressure sore, which was initially refractory, began to heal after starting treatment for SIBO. Among intrinsic factors related to the healing of pressure sores, blood levels of hemoglobin, albumin, and zinc are especially important [12]. Our patient had anemia, hypoalbuminemia, and a low zinc concentration, which gradually improved after starting treatment for SIBO.

SIBO is caused by multiple factors, including disturbances in defense mechanisms of the digestive tract, anatomical abnormalities, surgical interventions, and disturbed gastrointestinal motility [6, 10]. Bures et al. described several endogenous defense mechanisms that prevent bacterial overgrowth [4], including secretion of gastric acid, intestinal motility, a properly functioning ileocecal valve, production of secretory immunoglobulins on the surface of the gastrointestinal mucous membrane, and the bacteriostatic properties of pancreatic juice and bile. In our case, the patient’s history of cholecystectomy and hysterectomy were possible causes or exacerbating factors of SIBO. Disturbed gastrointestinal motility caused by paraplegia below the L4 level due to SCI is another possible cause of SIBO in our case, as well as decreased physical activity due to fracture of the left humerus and bilateral tibia.

Chronic gastrointestinal involvement is seen in 27–62% of patients with SCI [1, 13, 14]. SCI patients lack central nervous system control over the gastrointestinal system [15]. Liu et al. report that bowel problems in SCI patients are related to high levels of cord injury, completeness of cord injury, and post-injury durations of 10 years or more [2]. Moderate or severe grade depressive status is also associated with neurologic bowel dysfunction in SCI patients. Of these risk factors, our patient had complete cord injury that had occurred more than 10 years ago. Also, many bowel symptoms appear in patients with SCI (e.g., constipation, distension, incontinence, abdominal pain, bowel accidents, nausea, diarrhea, straining, rectal bleeding, hemorrhoids, autonomic hyperreflexia, headaches or sweat relieved by a bowel movement [2, 14, 16–21]). However, our patient showed no appetite loss, a sufficient amount of food intake, and non-severe bowel symptoms. Thus, the presence of malnutrition despite adequate food intake and low levels of lipid-soluble vitamins that were unresponsive to supplementation led us to suspect SIBO.

Although gastrointestinal symptoms are frequently observed in patients with SCI, there are few reports of SIBO in SCI patients. Cheng et al. report that 39% (145 of 377) of SCI patients were diagnosed with SIBO based on the glucose hydrogen/methane breath test [22]. However, the prevalence of SIBO among SCI patients as confirmed by the consensus diagnostic criteria of the presence of more than 10^5^ CFU/mL bacteria or any amount of *E. coli* in upper digestive tract content is unknown. In patients with SCI, absent central nervous system innervation of the digestive tract can change the inhabiting environment of bacteria. Gungor et al. report differences in gut microbial patterns between SCI patients and control individuals as measured by bacterial genome sequencing [15]. Specifically, they found that butyrate-producing bacteria were specifically reduced in SCI patients. Thus, it is possible that SIBO is overlooked in patients with SCI. In our case, it is unclear when SIBO occurred relative to the time of SCI, but we suspect that it arose due to gastrointestinal motility disorder caused by autonomic disturbances.

Disturbances in fat absorption and deficiency in fat-soluble vitamins (i.e., vitamins A, K, E, and D_3_) are observed in patients with SIBO [10]. Excess bacteria in the small intestine promotes a change from conjugated bile acid into deconjugated bile acid, which decreases the micellar solubilization of dietary fat. Bacterial fermented short chain fatty acid causes osmotic water movement to the intestinal lumen, which results in diarrhea and malabsorption [10]. Intestinal epithelial damage in SIBO also interferes with fat absorption. Mucosal damage is caused by metabolites of aerobic bacteria, endotoxins of anaerobic bacteria, and lithocholic acid, which is a bacterial degradation product of unconjugated bile acid [23–25]. Our patient, however, showed constipation rather than diarrhea in spite of SIBO. Whether or not diarrhea occurs in patients with SIBO is determined by multiple factors. Constipation frequently occurs in patients with SCI due to decreased physical activity and autonomic dysfunction. De Looze et al. reported that the rate of constipation in the patients with SCI is 58% [18]. A certain proportion of the patients with SCI show constipation in spite of the coexisting SIBO. Cheng et al. reported that in patients with both SCI and SIBO, 22% showed constipation [22]. We believe that the, factors leading to constipation in our patient were stronger than those leading to diarrhea. Vitamin D deficiency in SIBO causes osteomalacia. Our patient showed multiple fractures and osteoporosis with serum vitamin D_3_ levels below the detection limit and refractory to supplementation.

There is no consensus on the choice, dose, or duration of antibiotics for treating SIBO [6]. In principle, antibiotics should be chosen based on the results of an antimicrobial susceptibility test, but this approach cannot address the great diversity in microbiota of the digestive tract [26, 27]. Metronidazole is a first-line choice for SIBO [28], with other choices being rifaximin, ciprofloxacin, norfloxacin, amoxicillin/clavulanate, trimethoprim, sulfamethoxazole, cephalexin, or their combination [6]. However, these antibodies are selected based on custom rather than scientific evidence [27]. In our case, we used oral polymyxin B and amphotericin B in accordance with SDD, which was first reported as a method of preventing ventilation-associated pneumonia and microbial translocation of gram-negative rod bacteria and fungi in critically ill patients treated in the intensive care unit [29–31]. Polymyxin B administered to the digestive tract is non-absorbent into the human body and has strong bactericidal power against gram-negative rod bacteria except for naturally polymyxin-resistant bacteria such as *Proteus*, *Providencia*, *Morganella*, *Burkholderia*, and *Serratia* [32]. Amphoteric B is an antifungal drug that is also non-absorbent into the human body when administered to the digestive tract. In our case, after starting SDD, fat-soluble vitamins were increased, and osteoporosis was improved. No obvious adverse effects of SDD, such as antibiotic-associated diarrhea, were observed in our case.

When and how to stop antibiotherapy for the treatment of patients with SIBO are difficult problems. Few reports are available for the method and timing for making a decision to stop antibiotherapy in SIBO. Lauritano et al. reported that the recurrence rate at 9 months after stopping antibiotherapy in SIBO patients is 44% [33]. They also showed that an older age, history of appendectomy, and chronic use of PPIs are associated with SIBO recurrence. Bures et al. reported that cyclical gastrointestinal selective antibiotics are needed for SIBO treatment [4]. These reports indicate that in many patients with SIBO, it is actually impossible to stop antibiotherapy because of the underlying conditions that lead to SIBO. Similarly, in our case, it was difficult to ameliorate the underlying condition of decreased motility of the digestive tract due to SCI. We were compelled to continue SDD for a long duration. We did, however, succeed in gradually reducing the dose of polymyxin B and end the use of amphotericin B without signs of SIBO recurrence. With careful consideration, it may be possible and feasible to stop SDD completely.

Probiotics are also a treatment approach for SIBO, as some species of bacteria are thought to protect against high numbers of *E. coli* and fungi in the digestive tract [34]. However, the role and effects of probiotics are still unclear. The digestive tract microbiome has both pathogenic potential and a protective role in maintaining health. However, metagenomic analysis reveals that 20–60% of microorganisms in the digestive tract cannot be cultured under laboratory conditions [10]. The effects of SDD and probiotics on the digestive tract microbiome in patients with SIBO should be investigated to further understand the pathogenesis of the disease.

In conclusion, we treated a patient with a sacral pressure sore who also had SCI, multiple fractures with osteoporosis, and malabsorption, especially of fat-soluble vitamins. Based on culture of upper digestive tract content, we diagnosed the patient with SIBO and started SDD using polymyxin B and amphotericin B, which effectively ameliorated the absorbency disturbance and allowed healing of the pressure sore. In light of several common risk factors between pressure sores and SIBO, such as decreased physical activity, our case provides additional information on the associations among pressure sores, malnutrition, and SIBO.

## Data Availability

Data on this case not reported in the manuscript are available from the corresponding author upon reasonable request.

## Abbreviations

CFU: Colony forming units
SCI: Spinal cord injury
SDD: Selective decontamination of the digestive tract
SIBO: Small intestinal bacterial overgrowth
25(OH)VitD_3_: 25-hydroxy vitamin D_3_

## References

[1] Stone JM, Nino-Murcia M, Wolfe VA, Perkash I (1990). Chronic gastrointestinal problems in spinal cord injury patients: a prospective analysis. Am J Gastroenterol.

[2] Liu CW, Huang CC, Chen CH, Yang YH, Chen TW, Huang MH (2010). Prediction of severe neurogenic bowel dysfunction in persons with spinal cord injury. Spinal Cord.

[3] Eglseer D, Hodl M, Lohrmann C (2019). Nutritional management of older hospitalised patients with pressure injuries. Int Wound J.

[4] Bures J, Cyrany J, Kohoutova D, Forstl M, Rejchrt S, Kvetina J, Vorisek V, Kopacova M (2010). Small intestinal bacterial overgrowth syndrome. World J Gastroenterol.

[5] Matsui Y, Furue M, Sanada H, Tachibana T, Nakayama T, Sugama J, Furuta K, Tachi M, Tokunaga K, Miyachi Y (2011). Development of the DESIGN-R with an observational study: an absolute evaluation tool for monitoring pressure ulcer wound healing. Wound Repair Regen.

[6] Sachdev AH, Pimentel M (2013). Gastrointestinal bacterial overgrowth: pathogenesis and clinical significance. Ther Adv Chronic Dis.

[7] Groah SL, Schladen M, Pineda CG, Hsieh CH (2015). Prevention of pressure ulcers among people with spinal cord injury: a systematic review. PM R.

[8] Vantrappen G, Janssens J, Hellemans J, Ghoos Y (1977). The interdigestive motor complex of normal subjects and patients with bacterial overgrowth of the small intestine. J Clin Invest.

[9] Zoetendal EG, Raes J, van den Bogert B, Arumugam M, Booijink CC, Troost FJ, Bork P, Wels M, de Vos WM, Kleerebezem M (2012). The human small intestinal microbiota is driven by rapid uptake and conversion of simple carbohydrates. ISME J.

[10] Miazga A, Osinski M, Cichy W, Zaba R (2015). Current views on the etiopathogenesis, clinical manifestation, diagnostics, treatment and correlation with other nosological entities of SIBO. Adv Med Sci.

[11] Khoshini R, Dai SC, Lezcano S, Pimentel M (2008). A systematic review of diagnostic tests for small intestinal bacterial overgrowth. Dig Dis Sci.

[12] Heintschel M, Heuberger R (2017). The potential role of zinc supplementation on pressure injury healing in older adults: a review of the literature. Wounds.

[13] Han TR, Kim JH, Kwon BS (1998). Chronic gastrointestinal problems and bowel dysfunction in patients with spinal cord injury. Spinal Cord.

[14] Ebert E (2012). Gastrointestinal involvement in spinal cord injury: a clinical perspective. J Gastrointestin Liver Dis.

[15] Gungor B, Adiguzel E, Gursel I, Yilmaz B, Gursel M (2016). Intestinal microbiota in patients with spinal cord injury. PLoS One.

[16] Harari D, Sarkarati M, Gurwitz JH, McGlinchey-Berroth G, Minaker KL (1997). Constipation-related symptoms and bowel program concerning individuals with spinal cord injury. Spinal Cord.

[17] Menter R, Weitzenkamp D, Cooper D, Bingley J, Charlifue S, Whiteneck G (1997). Bowel management outcomes in individuals with long-term spinal cord injuries. Spinal Cord.

[18] De Looze D, Van Laere M, De Muynck M, Beke R, Elewaut A (1998). Constipation and other chronic gastrointestinal problems in spinal cord injury patients. Spinal Cord.

[19] Lynch AC, Wong C, Anthony A, Dobbs BR, Frizelle FA (2000). Bowel dysfunction following spinal cord injury: a description of bowel function in a spinal cord-injured population and comparison with age and gender matched controls. Spinal Cord.

[20] Krogh K, Nielsen J, Djurhuus JC, Mosdal C, Sabroe S, Laurberg S (1997). Colorectal function in patients with spinal cord lesions. Dis Colon Rectum.

[21] Chen CY, Chuang TY, Tsai YA, Tai HC, Lu CL, Kang LJ, Lu RH, Chang FY, Lee SD (2004). Loss of sympathetic coordination appears to delay gastrointestinal transit in patients with spinal cord injury. Dig Dis Sci.

[22] Cheng X, Zhang L, Xie NC, Xu HL, Lian YJ (2017). Association between small-intestinal bacterial overgrowth and deep vein thrombosis in patients with spinal cord injuries. J Thromb Haemost.

[23] Kirsch M, Bozdech J, Gardner DA (1990). Hepatic portal venous gas: an unusual presentation of Crohn's disease. Am J Gastroenterol.

[24] Jones RM, Neish AS (2011). Recognition of bacterial pathogens and mucosal immunity. Cell Microbiol.

[25] Hoog CM, Lindberg G, Sjoqvist U (2007). Findings in patients with chronic intestinal dysmotility investigated by capsule endoscopy. BMC Gastroenterol.

[26] Singh VV, Toskes PP (2004). Small bowel bacterial overgrowth: presentation, diagnosis, and treatment. Curr Treat Options Gastroenterol.

[27] Quigley EM, Abu-Shanab A (2010). Small intestinal bacterial overgrowth. Infect Dis Clin N Am.

[28] Melchior C, Gourcerol G, Bridoux V, Ducrotte P, Quinton JF, Leroi AM (2017). Efficacy of antibiotherapy for treating flatus incontinence associated with small intestinal bacterial overgrowth: a pilot randomized trial. PLoS One.

[29] Vandenbroucke-Grauls CM, Vandenbroucke JP (1991). Effect of selective decontamination of the digestive tract on respiratory tract infections and mortality in the intensive care unit. Lancet.

[30] Silvestri L, van Saene HK, Casarin A, Berlot G, Gullo A (2008). Impact of selective decontamination of the digestive tract on carriage and infection due to gram-negative and gram-positive bacteria: a systematic review of randomised controlled trials. Anaesth Intensive Care.

[31] Camus C, Salomon S, Bouchigny C, Gacouin A, Lavoue S, Donnio PY, Javaudin L, Chapplain JM, Uhel F, Le Tulzo Y (2014). Short-term decline in all-cause acquired infections with the routine use of a decontamination regimen combining topical polymyxin, tobramycin, and amphotericin B with mupirocin and chlorhexidine in the ICU: a single-center experience. Crit Care Med.

[32] Olaitan AO, Morand S, Rolain JM (2014). Mechanisms of polymyxin resistance: acquired and intrinsic resistance in bacteria. Front Microbiol.

[33] Lauritano EC, Gabrielli M, Scarpellini E, Lupascu A, Novi M, Sottili S, Vitale G, Cesario V, Serricchio M, Cammarota G (2008). Small intestinal bacterial overgrowth recurrence after antibiotic therapy. Am J Gastroenterol.

[34] Reuben RC, Roy PC, Sarkar SL, Alam RU, Jahid IK (2019). Isolation, characterization, and assessment of lactic acid bacteria toward their selection as poultry probiotics. BMC Microbiol.

